# Interpreting the Consequences of Metformin Accumulation in an Emergency Context: Impact of the Time Frame on the Blood Metformin Levels

**DOI:** 10.1155/2014/717198

**Published:** 2014-12-17

**Authors:** Jean-Daniel Lalau, Farshad Kajbaf

**Affiliations:** ^1^Service d'Endocrinologie-Nutrition, Hôpital Sud, 80054 Amiens, France; ^2^INSERM U-1088, Université de Picardie Jules Verne, 80037 Amiens, France

## Abstract

*Objective*. To clarify the link between metformin accumulation and its metabolic consequences by taking the time frame for metformin measurement into account. *Research Design and Methods*. Our database was studied for cases of metformin accumulation and lactic acidosis status available on admission, and then we selected patients in whom arterial pH, blood lactate, and plasma and erythrocyte metformin levels had been determined at the same time point. *Results*. Seventeen reports were studied on 16 patients, of whom 10 presented lactic acidosis. The time interval between admission and comprehensive testing ranged from 0 to 52 hours. The study parameters were determined simultaneously on admission in only 4 patients. In the 9 patients with lactic acidosis on admission and a delayed metformin assay, lactic acidosis persisted in 6 cases and had resolved in 3 cases by the time the blood sampling for metformin assay was performed. Conversely, lactic acidosis developed after admission in one case. *Conclusions*. Caution must be taken when interpreting the consequences of metformin accumulation in an emergency context: the patient's lactic acidosis status will have changed by the time the metformin assay is performed, even though metformin accumulation may still be present.

## 1. Introduction

It is well known that there is a relationship between metformin levels and lactate metabolism: the higher the blood metformin concentration, the more severe the metabolic disturbance [1, 2]. However, the relationship between metformin and lactic acidosis is more complex because (i) lactic acidosis is not necessarily accompanied by metformin accumulation in metformin-treated patients; (ii) metformin accumulation does not necessarily lead to lactic acidosis; and, conversely, (iii) metformin may induce lactic acidosis in the absence of drug accumulation [3].

Studying the relationship between metformin and lactic acidosis therefore involves taking account of putative confounding factors [4], such as the patient profile, the disease context, the assay methods, the type of metformin accumulation (acute versus chronic), the time of the last metformin administration, the large interindividual variations in blood metformin levels for a given therapeutic dose, and, lastly, interindividual variations in the metabolic response to metformin accumulation [5, 6]. Surprisingly, the time frame for determining the status of metformin accumulation has not previously been considered as a confounding factor, even though a delay in measuring blood metformin levels is largely unavoidable in an emergency context in which a metformin assay is not the top priority. Indeed, the late performance of a blood metformin assay may underestimate the level of drug present in the patient on admission (which should be followed by metformin's withdrawal as a result of emergency admission), especially when the patient is then treated with dialysis and/or the administration of vasoactive drug. Furthermore, the latter procedures may also rapidly modify the blood lactate concentration.

We therefore sought to clarify the link between metformin accumulation and its metabolic consequences by taking the time frame for metformin measurement into account and test the following hypothesis: the patient's lactic acidosis status will have changed by the time the metformin assay is performed.

Accordingly, we recorded (i) the proportion of patients for whom the metformin assay was delayed after their admission; (ii) the mean (range) of the time interval between the determination of lactic acidosis on admission and the metformin measurement; and (iii) the impact of the time interval between these two determinations on the assessment of the patient's metabolic status.

## 2. Research Design and Methods

### 2.1. Data Source

We systematically reviewed all the blood metformin assay data recorded by our hospital's laboratory between January 2004 and December 2013. In general, metformin assays had been requested in order to adjust the dose to the patient's renal status or to screen metformin accumulation. We then selected for analysis all patients with metformin accumulation (defined as a plasma metformin concentrations ≥ 5 mg/L, corresponding to 10 times the mean reported therapeutic value [7]) and with available lactic acidosis status on admission.

### 2.2. Selection and Presentation of the Study Parameters

On the basis of the reports on patients with metformin accumulation, we noted the pH, lactate, and the plasma and erythrocyte metformin concentrations. We selected patients in whom all these parameters had been determined at the same time point during hospitalization. The data were then analysed for (i) lactic acidosis (arterial pH < 7.35 and a blood lactate concentration > 5 mmol/L [8]) on admission and at the time of the metformin assay and (ii) the extent of metformin accumulation.

## 3. Results

We identified 17 eligible reports on 16 patients aged between 44 and 74. For each individual, the clinical setting, arterial pH, arterial lactate, serum creatinine and blood metformin values, and the time interval between admission and subsequent assays are presented in Table 1 (cases 5 and 9 concern two different episodes in the same patient). All patients had hyperlactatemia (with a minimal value of 3.3 mmol/L) and 10 patients had lactic acidosis. At least one factor for hyperlactatemia associated with metformin accumulation was identified in all but one case. Kidney disease and sepsis appeared to be the main factors associated with metformin accumulation. The serum creatinine concentration was available on admission for 14 cases and was abnormal in 13 of them. Dialysis and/or the administration of vasoactive drugs were initiated prior to blood metformin determination in four and six patients, respectively.

Arterial pH and lactate were available for all 16 patients on admission. Eleven of the latter had acidosis. The study parameters were determined simultaneously on admission in only 4 patients. The time interval between the assessment of lactic acidosis status on admission and sample collection for the full set of parameters varied greatly from one patient to another; it was between 0 and 6 hours in eight cases, between 6 and 12 hours in five cases, between 12 and 24 hours in one case, between 1 and 2 days in two cases, and over 3 days (52 hours, in fact) in one case. The mean time interval was 12.5 ± 16 hours. In the 9 cases with lactic acidosis on admission and for whom the sampling for the metformin measurement was performed later, lactic acidosis persisted at the time of such measurement in 6 cases and had resolved in 3 cases. Conversely, one patient (case 9) developed lactic acidosis after admission.

Blood metformin levels were high in all reports, even after dialysis and even for the longest time to blood sampling, and ranged from 6.3 to 66.8 mg/L in plasma (mean ± SD: 28.7 ± 21.3; normal upper limit <1.35) and 1.5 to 20.1 mg/L in erythrocytes (mean ± SD: 12.3 ± 6.6; normal upper limit <1.65). The plasma metformin levels were higher than the erythrocyte metformin levels whenever the time to blood sampling after admission was below 8 hours and in half of the cases with a longer time interval.

Lastly, three of the 16 patients (18.8%) failed to survive.

## 4. Discussion

The present study is the first to document patients with marked metformin accumulation as a function of the time frame for blood sampling. Lactic acidosis was observed in 10 out of the 17 reports in our series.

In view of the issues highlighted in the Introduction, the following observations may be made: (i) in most cases (13 out of 17), the pH, lactate, and metformin values were not determined simultaneously on admission; (ii) the time interval between the initial sampling (for pH and lactate) and the subsequent sampling for obtaining other study parameters (including the metformin level) was generally rather short (about 12 hours) but varied markedly when considered the SD (16 hours) and especially the range (up to 52 hours); most importantly, (iii) the lactic acidosis status (i.e., resolution or occurrence) had changed in half of the patients for whom blood metformin levels had been determined with some delay.

It is also noteworthy that the three patients with the longest time interval between admission and subsequent sampling (about two days) still had very high plasma and erythrocyte metformin levels. At the time of the metformin measurement, the pH and lactate values no longer met the criteria for lactic acidosis. Although a high erythrocyte metformin concentration was expected (because of the drug's long half-life in these cells [9]), the persistent, high plasma concentration is surprising, especially since dialysis had been initiated in two of the three patients.

This study shares the limitations of all descriptive studies. However, it is fair to say that a trial limitation may be strength when it reflects what actually happens in clinical practice. More precisely, the present study's main objective was not to rigorously study the effect of metformin accumulation on lactate metabolism but rather to examine possible pitfalls when considering the link between metformin and so-called “metformin-associated lactic acidosis” under emergency conditions. In other words, we wanted to know which factors have an effect on this link, such as the time frame for blood sampling for pH, lactate, and metformin measurements.

There are two levels of complexity in this context: factors that modify the metformin level (such as the time to sampling after admission, in particular) and the physiopathological mechanism complexity of so-called “metformin-associated lactic acidosis” in itself [3]. Indeed, metformin accumulation may be related to either acute, primary renal failure or isolated metformin intoxication [10] (which occurred in less than one-third of the patients studied here) or secondary to renal failure as a complication of a severe, systemic disease (such as sepsis and heart failure) [11, 12]. For this reason, the existence of the term “metformin-associated lactic acidosis” does not imply that all the associated factors (e.g., the degree of metformin accumulation and the severity of any associated diseases) are present to the same degree at any given time.

Given the above results and considerations, our results suggest that when the time interval between the first lactate measurement and that of blood metformin is very long, caution must be taken when considering the putative relationship between metformin accumulation and lactic acidosis. Indeed, the patient's lactic acidosis status will have changed in some cases by the time the metformin assay is performed, even though metformin accumulation may still be present.

## 5. Conclusion

In patients with metformin accumulation, metformin levels are generally measured many hours after admission. Because of this delay, the patient's metabolic status (i.e., the presence or the absence of lactic acidosis) had changed in half of the cases studied here, even while metformin accumulation persisted. This delay should be taken into consideration when classifying patient with so-called “metformin-associated lactic acidosis.”

## Figures and Tables

**Table 1:** Individual patient data and clinical settings, listed according to the time interval between sets of blood measurements. CKD: chronic kidney disease.

Case	Clinical features			Time interval after admission, hours	Biochemical features on and after admission					
	Clinical setting	Dialysis (d) and/or vasoactive drugs (v)	Survival		Serum creatinine *μ*mol/L	pH	Arterial lactate mmol/L	Lactic acidosis status	Plasma metformin mg/L, *N* < 1.35	Erythrocyte metformin mg/L, *N* < 1.65
All study parameters immediately available on admission										

1	Acute aggravation of chronic heart failure	—	Yes	0	564	7.40	3.3	No lactic acidosis	9.7	4.8

2	No overt failure	—	Yes	0	—	7.40	5.4	No lactic acidosis	7.38	4.01

3	Sepsis	—	Yes	0	296	7.37	7.6	No lactic acidosis	7.6	2.53

4	Mesenteric ischemia, CKD	—	Yes	0	738	6.73	8.4	Lactic acidosis	66.8	20.1

Study parameters available partly on admission and completed some hours later										

5^*^	Multiple drug overdose	d	No	0	187	6.84	27	Lactic acidosis	ND	ND
				3	226	7.15	30	Persistence	56.7	18.6

6	Acute renal failure	—	Yes	0	776	7.20	20	Lactic acidosis	ND	ND
				4	745	7.23	18	Persistence	32.5	19

7	Acute aggravation of CKD	v	No	0	653	6.90	16	Lactic acidosis	ND	ND
				4	640	6.80	21	Persistence	43.3	8.7

8	Sepsis	v	No	0	161	6.70	28	Lactic acidosis	ND	ND
				5	160	6.67	26	Persistence	7.1	1.5

9^*^	Multiple drug overdose	v	Yes	0	68	7.41	6.6	No lactic acidosis	ND	ND
				7.5	127	7.17	11.4	Appearance	64	19

10	Acute renal failure	d	Yes	0	1073	7.26	3.7	No lactic acidosis	ND	ND
				8.5	486	7.41	1.6	No lactic acidosis	15.8	19.4

11	Pulmonary embolism and CKD	—	Yes	0	292	7.36	5.2	No lactic acidosis	ND	ND
				9	305	7.31	4.6	No lactic acidosis	20.5	13.2

12	Acute renal failure	—	Yes	0	ND	7.31	10.5	Lactic acidosis	ND	ND
				11	538	7.14	14.4	Persistence	38	8.7

13	Sepsis	v	Yes	0	1327	7.02	10.8	Lactic acidosis	ND	ND
				11.5	1285	7.21	7.6	Persistence	50.1	18.5

14	Sepsis	—	Yes	0	231	7.45	6	No lactic acidosis	ND	ND
				17	244	7.39	1.7	No lactic acidosis	6.3	7.6

15	Acute renal failure	—	Yes	0	ND	7.20	5.7	Lactic acidosis	ND	ND
				39	928	ND	4.1	Disappearance	10.4	15.1

16	Sepsis	dv	Yes	0	958	6.91	16	Lactic acidosis	ND	ND
				40	353	7.42	1.7	Disappearance	39.9	16.8

17	Metformin overdose and acute renal failure	dv	Yes	0	1357	6.79	20	Lactic acidosis	ND	ND
				52	ND	7.36	2.1	Disappearance	11.4	11.3

^*^Cases 5 and 9 concern the same patient.
